# Unambiguous Characterization of *p*-Cresyl Sulfate, a Protein-Bound Uremic Toxin, as Biomarker of Heart and Kidney Disease

**DOI:** 10.3390/molecules24203704

**Published:** 2019-10-15

**Authors:** Rita Paroni, Silvana Casati, Michele Dei Cas, Monica Bignotto, Federico Maria Rubino, Pierangela Ciuffreda

**Affiliations:** 1 Dipartimento di Scienze della Salute, Università degli Studi di Milano, Via Di Rudinì, 8, 20142 Milano, Italy; rita.paroni@unimi.it (R.P.); michele.deicas@unimi.it (M.D.C.); monica.bignotto@unimi.it (M.B.); federico.rubino@unimi.it (F.M.R.); 2 Dipartimento di Scienze Biomediche e Cliniche “Luigi Sacco”, Università degli Studi di Milano, Via G.B. Grassi 74, 20157 Milano, Italy; silvana.casati@unimi.it

**Keywords:** *p*-cresyl sulfate, chronic kidney disease (CKD), urine, mass spectrometry, HPLC, bioanalysis

## Abstract

*p*-Cresyl sulfate is one of the bound uremic toxins whose level increases in the sera of patients with the severity of chronic kidney disease and is therefore used as a standard for clinical investigations. Our first attempts to obtain *p*-cresyl sulfate led exclusively to the product of sulfonation of the aromatic ring instead of sulfation on the OH moiety. Nevertheless, this initial discouraging result allowed us to handle both *p*-cresyl sulfate and 2-hydroxy-5-methylbenzenesulfonic acid obtained by different synthetic pathways. Interestingly, the comparison between the two isomers pointed out that the two molecules show the same fragmentation pattern and are indistinguishable by mass spectrometry. They cannot be separated on several commercially available columns. The only difference between the two compounds is a 10-fold higher ionization yield under negative ion electrospray ionization. NMR spectral studies definitely confirmed the different molecular structures. We present here an unambiguous biomimetic synthetic route for *p*-cresyl sulfate and the spectroscopic characterization of both the compounds by nuclear magnetic resonance and mass spectrometry.

## 1. Introduction

The aryl sulfates, *p-*cresyl sulfate (*p*CS) and indoxyl sulfate (IxS), are protein-bound uremic toxins (UTx), the levels of which increase in the sera of patients with the severity of chronic kidney disease (CKD), and both have been shown to have a strong negative correlation with renal function (estimated glomerular filtration rate (eGFR)) [1,2,3,4]. Toxicity of *p*CS has been associated with a number of clinical conditions [5] that show an increase in *p*CS, such as children autism [6] and heart diseases [7].

*p-*Cresol is both a minor biotransformation product of toluene, a volatile organic compound (VOC) present in urban atmosphere and an industrial solvent, and a metabolite of anaerobic bacteria that biotransform the amino acids tyrosine and phenylalanine [8]. According to current knowledge, *Clostridium difficile*, a major cause of diarrhea, is one of the main producers of *p*CS in the human gut [9]. At the molecular scale, the key process of an enzymatic multistage mechanism is degradation of *p*-hydroxyphenyl-2-keto-propionic acid, the Tyr transamination catabolite, to *p*CS by 4-hydroxyphenylacetate decarboxylase (EC 4.1.1.83), an [FeS] enzyme [5], whose active site entails the presence of a glycyl radical enzyme [10]. Another pathway may entail *p-*hydroxyphenylpyruvic acid as the direct precursor of *p-*cresol, through the action of another glycyl radical enzyme [11].

**Figure 1:**
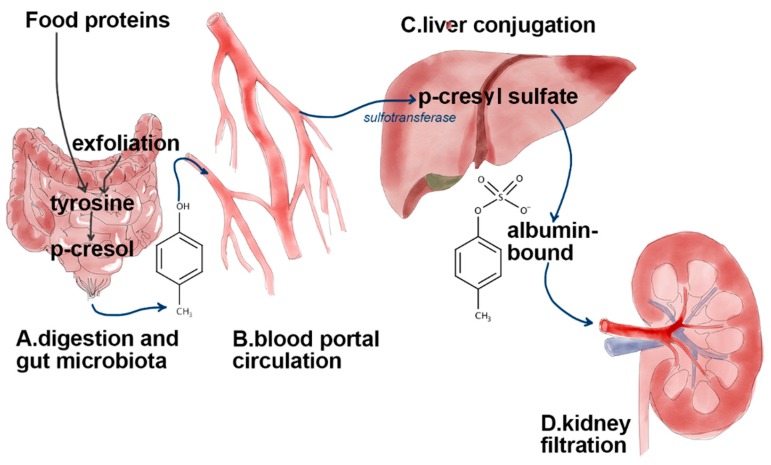
Graphical overview of biochemical transformation of tyrosine coming from food proteins and cellular exfoliation in gut by *Clostridium difficile.* Through portal circulation, *p*-cresol reaches the liver where it is bio-transformed by sulfotransferase into *p*-cresyl sulfate. In healthy persons, more than 99% of *p*CS is bound to albumin, thus excretion through the kidney occurs primarily via tubular secretion by an organic anion transport system. This binding is decreased in uremic patients.

In humans, hepatic phase II biotransformation converts the phenolic products into their glucuronide and sulfate end products for urinary elimination [12,13,14] (Figure 1). Due to their strong binding to plasma proteins, both *p*CS and IxS cannot be removed efficiently by hemodialysis (HD) or peritoneal dialysis (PD) [15] and accumulate, thus their measurement in plasma can be used to monitor the extent of organ damage in CKD patients undergoing HD or PD and awaiting kidney transplantation. Although analytical methods that employ pre-column and liquid chromatography with fluorescence detection have been developed to establish reference ranges of IxS and *p*CS [16], a reliable analytical method employing mass spectrometry needs the availability of authenticated pure standards and of isotope-labeled internal standards for robust quantification [16].

When we started this work, *p*CS was hardly commercially available, so we decided to prepare and characterize authentic specimens for method set-up and validation. Synthetic approaches were tested; however, the results were different from those anticipated from the literature [16,17,18,19,20,21,22,23]. The necessity of unambiguous product characterization and reliable route for *p*CS sample preparation highlighted some novel facets of this deceivingly simple molecule.

## 2. Results and Discussion

### 2.1. Compound Preparation

During our studies of simultaneous quantification of the UTx compounds, *p*CS and IxS, we needed to synthetize authentic *p*CS in its natural abundance and isotopically labeled forms.

For the synthesis of *p*CS (**1**), we started from the method [23] used by a number of authors [16,17,18,19,20,21,22]. Unfortunately, in our hand, this procedure [21,23] led exclusively to the sulfonation of the aromatic ring (**2**) instead of sulfation on the OH moiety (**1**).

The elucidation of real structure of the obtained compound was achieved by a careful study of ^1^H-NMR and ^13^C-NMR spectra (Section 2.2) that let us to conclude that the compound was 2-hydroxy-5-methylbenzenesulfonic acid (**2**) rather than *p*CS (**1**). To our knowledge, a detailed description of NMR spectra of **1** and **2** has not been reported yet and for this reason, we thought it useful to underline this topic.

A careful analysis of the literature [24,25,26,27,28,29] shows how obtaining the sulfation product could be a challenging target, due to the concurrent formation of the sulfonation product. In particular, Cerfontain and Koeberg-Telder [28] reported a detailed study of the sulfonation of 4-methylphenol in H_2_SO_4_ ranging in concentration from 81.6–112.2% and noted that the 2- to 3-sulfonic acid ratio increases strongly upon increasing the H_2_SO_4_ concentration. The ^1^H-NMR spectrum is also reported.

**Figure 2:**
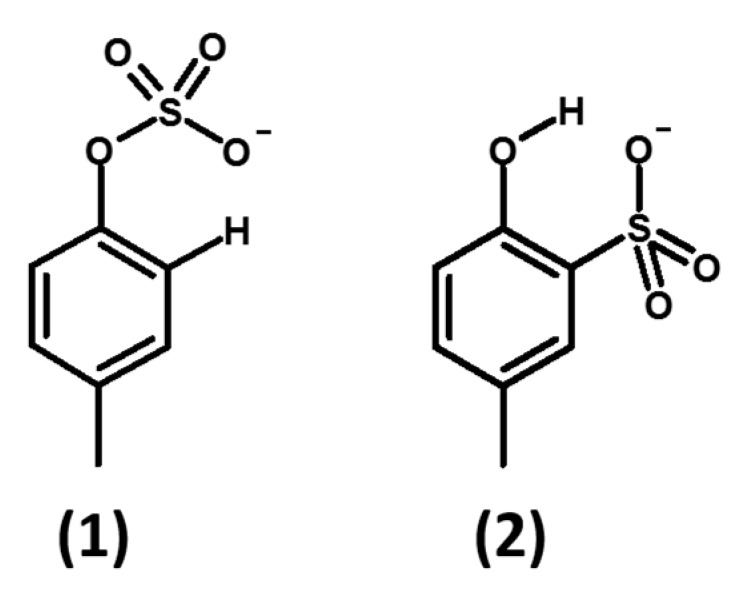
Structure of reaction products of activated sulfuric acid at the oxygen atom (sulfation, **1**), at a ring carbon atom (sulfonation, **2**).

The first attempts made by us in accordance with literature [23,30] with chlorosulfonic acid as the source of sulfate and dimethyl aniline or pyridine as the in-situ base and leaving group, gave the exclusive formation of the sulfonated product (**2**) (Figure 2), by reaction at the *ortho*-ring carbon atom. Changing the sequence of reagents, the reaction temperature, and the base did not give the expected result [31]. Direct sulfation with sulfuric acid at different concentrations gave only the sulfonation product (**2**).

After a careful investigation about the reaction conditions, we managed to obtain target compound **1**, only through a condensation procedure with dicyclohexylcarbodiimide (DCC). This improved procedure is serendipitously biomimetic, since it entails the use of an activated, electrophilic form of sulfuric acid, in principle analogous to 3′-phosphoadenosine-5′-phosphosulfate, which is the active cofactor of sulfokinases (SULT), the enzymes responsible for sulfate transfer to phenolic and alcohol-bearing phase I biotransformation products of endogenous and xenobiotic substrates [32]. This may explain the success of the condensation method in avoiding the concomitant formation of sulfonic acid, which is the only product when sulfuric acid or chlorosulfonic acid are employed.

### 2.2. Compound Characterization

#### 2.2.1. NMR Analysis

The structural assignment of the products were made by ^1^H-NMR spectroscopy and were based on the relative area ratios of the peaks, the multiplicity of various signals, the coupling constants, and the specific substituent shielding parameters [33,34].

**Figure 3:**
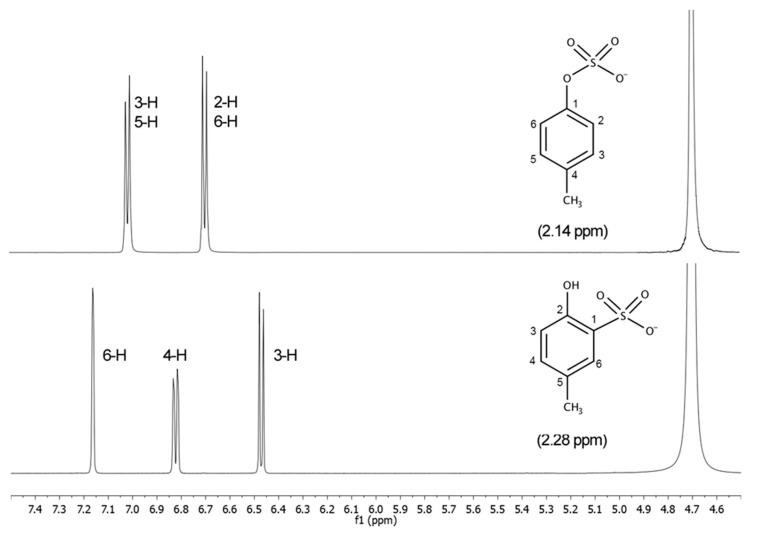
^1^H-NMR spectra of *p*CS (**1**, **top**) and 2-hydroxy-5-methylbenzenesulfonic acid (**2**, **bottom**).

**Figure 4:**
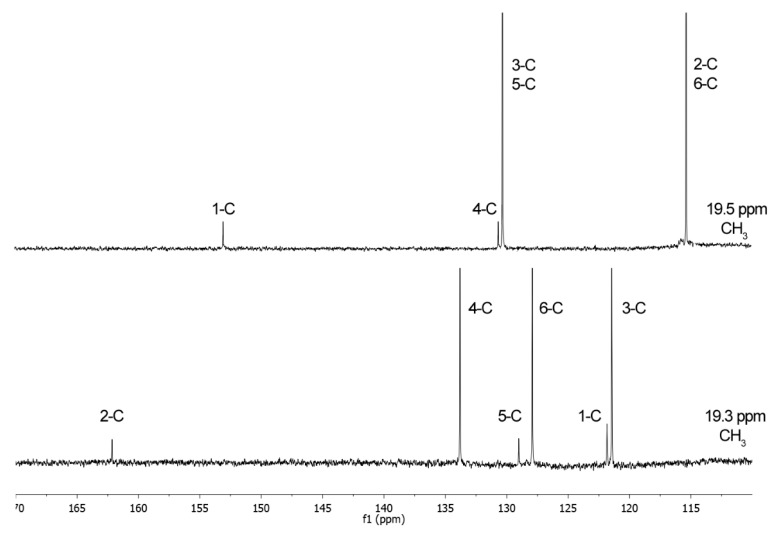
^13^C-NMR spectra of *p*CS (**1**, **top**) and 2-hydroxy-5-methylbenzenesulfonic acid (**2**, **bottom**).

Figure 3 and Figure 4 show the ^1^H-NMR and the ^13^C-NMR spectra, respectively, of *p*CS (**1**, top) and 2-hydroxy-5-methylbenzenesulfonic acid (**2**, bottom). The important differences in the spectra allow unequivocal identification of the individual isomers, either after purification or directly in a reaction mixture. In the ^1^H-NMR spectrum of compound **2** initially obtained, there are three different kinds of hydrogens on the aromatic ring at 7.47, 7.13, and 6.78 ppm instead of the expected two. In *p*CS to a simple first order approximation, the appearance of the signals for all four ^1^H-atoms are readily predictable. The H-2 and H-6 are equivalent and so produce one chemical shift just like the H-3 and H-5 at 6.71 and 7.03 ppm, respectively.

The same applies to ^13^C-NMR spectrum showing six different peaks, three substituted aromatic carbon atoms at 133.8, 127.9, and 121.4 ppm, and three unsubstituted carbon atoms at 162.1, 129.0, and 121.8 ppm, thereby demonstrating that in the obtained compound there are six different carbon environments at 162.1, 133.8, 129.0, 127.9, 121.8, and 121.4 ppm. Instead, in *p*CS (**1**), there are four different carbon environments and four different peaks. These considerations got us thinking that, in our hands, applying the method reported by Feigenbaum et al. [23], the sulfonation (reaction at a ring carbon atom) took place instead of sulfation (reaction at the oxygen atom).

#### 2.2.2. Mass Spectrometry

Characterization and differentiation of the isomers **1** and **2** by mass spectrometry proved much more challenging, in an unexpected way. Our negative ion ESI-MS/MS spectra of NMR-characterized *p*CS (**1**) are comparable to that reported by Attygalle et al. [35] for the lower homolog phenyl sulfate and show the expected fragmentation channels (Supplemental Material, Figure S1).

**Figure 5:**
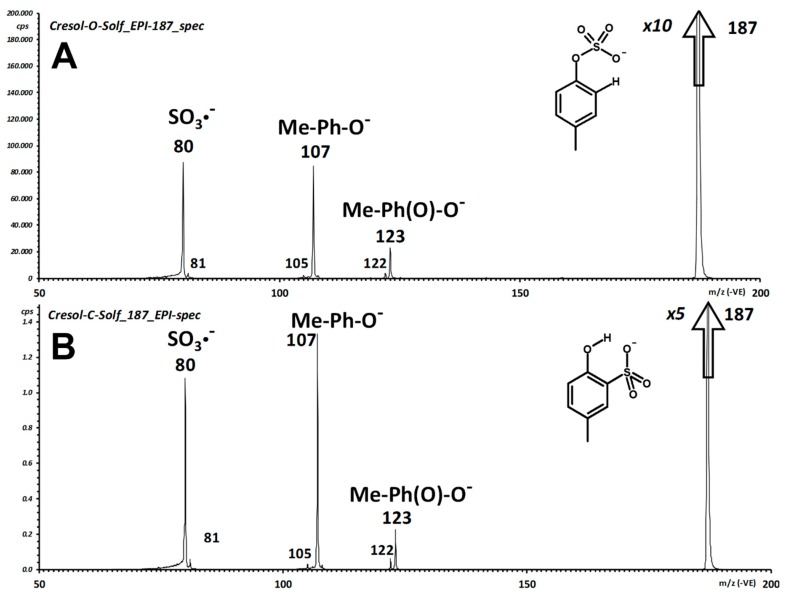
Integrated fragment spectrum of *p*CS (**1**; **A**) and 2-hydroxy-4-methyl-phenylsulfonic acid (**2**; **B**).

Essentially the same expected reaction pathways occur for the two compounds, with the only difference of the ratio of the main ion signals (Figure 5). The fragmentation pathways are those expected, and entail generation of deprotonated *p-*cresol (*m/z* 107) and of electron-attached sulfur trioxide (SO_3_^−^^●^; *m/z* 80 for ^32^S, 82 for ^34^S).

In *p*CS (**1**), a weak, yet intriguing transition leads to oxygen insertion and formation of deprotonated 4-methyl-1,2-dihydroxy-benzene (*m/z* 123), the connectivity of which is supported by observation of H^●^ loss to yield the radical anion of 4-methyl*-o-*quinone (*m/z* 122). One main objective of our extensive characterization by mass spectrometry of the isomers **1** and **2** is to try to discriminate the two possible connectivities, i.e., the phenyl sulfate from the aryl sulfonic acid. As apparent from the pair of displayed integrated fragment spectra (Figure 5), identical fragmentation is obtained for the two isomers.

In addition, unimolecular decomposition of the deprotonated molecule (*m/z* 187) of both compounds yields almost superimposable breakdown curves of the main fragment ions (Supplemental Material, Figure S2). A detailed discussion of the fragmentation processes of the two compounds is reported as Supplemental Material (Figure S3) and references therein.

### 2.3. Studies for Separation of pCS (***1***) and 2-Hydroxy-5-Methylbenzenesulfonic Acid (***2***) by LC-MS/MS

**Figure 6:**
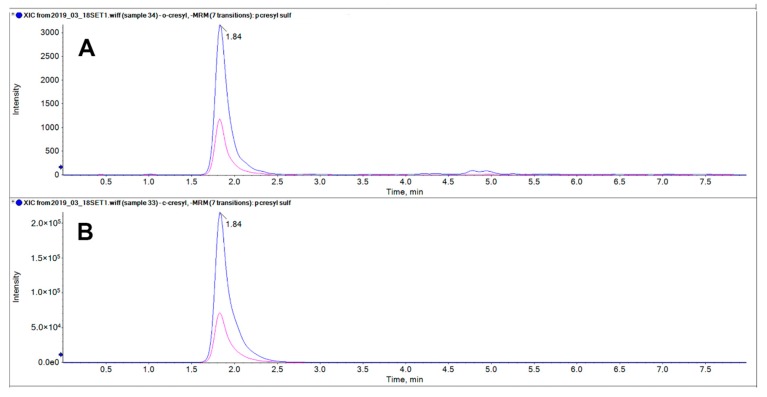
LC-MS/MS analysis of 25 μM *p*CS (**1**, **A**) and 25 μM 2-hydroxy-5-methylbenzenesulfonic acid (**2**, **B**). The pink and the blue transition correspond respectively to *m/z* 187.1 > 80 and 187.1 > 107. The ion yield of isomer B is close to 100-fold-higher than that of isomer A.

The optimization of analysis conditions was undertaken in order to ensure a reliable separation of *p*CS (**1**) and 2-hydroxy-5-methylbenzenesulfonic acid (**2**). Several column phases were tested such as C18, C18-amide, C18-polar, ether-linked, and hydrophilic interaction liquid chromatography. None of them provided the desired separation, and neither did pentafluorophenyl phase coupled to the use of ammonium buffer and 0.01% of formic acid, which instead allowed a baseline separation of *p*CS and IxS (data not shown). The only difference showed between *p*CS (**1**) and 2-hydroxy-5-methylbenzenesulfonic acid (**2**) was the intensity displayed when they were injected at the same concentration: singularly **2** was 10-fold-higher than **1** (Figure 6).

## 3. Material and Methods

### 3.1. Chemistry

Chemicals, solvents, and standards were obtained from Sigma-Aldrich and used without further purification. The progress of the reactions was monitored by analytical thin-layer chromatography (TLC) on pre-coated glass plates (silica gel 60 F254-plate-Merck, Darmstadt, Germany) and the products were visualized by UV light. Purity of all compounds (>99%) was verified by thin-layer chromatography and NMR measurements [36,37].

### 3.2. Synthesis of p-Cresyl Sulfate (***1***)

DCC (4 g, 21 mmol) in dichloromethane anhydrous was added to a cold solution of *p*-cresol (1.5 g, 14 mmol) in dichloromethane anhydrous. After 20 min under stirring at 0 °C, concentrated sulfuric acid (800 μL, 14 mmol) was slowly added and stirring continued for 2 h at room temperature. The reaction was monitored by TLC (CH_2_Cl_2_/MeOH 95:5, *v/v*). NaOH was added and the precipitate was filtered under vacuum, washed with dichloromethane, solubilized in methanol, and concentrated. Cold diethyl ether was added giving a white precipitate corresponding to the desired product as sodium salt (1 g, 4.8 mmol, 34%). The title compound was obtained by treatment with acid under mild conditions: 210 mg (1 mmol) of the sodium salt was dispersed in methanol (2 mL) under stirring, then an acidic resin (10% *w/w*; Dowex 50WX8-200) was added. When the compound was completely dissolved (1 h), the product was recovered by filtration under vacuum and subsequent evaporation of the solvent (yield >98%); mp. 250 °C (sample turns to brown), 284 °C (decomposition).

### 3.3. 2-Hydroxy-5-methylbenzenesulfonic Acid (***2***) Formation

#### 3.3.1. General Procedure

1.2 g of chlorosulfonic acid (10 mmol) were added dropwise with constant stirring to an ice-cooled solution of 1g of *p*-cresol (9.25 mmol) in 10 mL of pyridine. After 1 h, NaOH was added and the precipitate was recovered by filtration under vacuum. The solid was solubilized in methanol and reprecipitated with cold diethyl ether giving the sodium salt of compound **2** as a white solid 1.6 g (8.32 mmol, 90%). The same reaction was also performed at −78 °C. The title compound was obtained by treatment with acid under mild conditions.

#### 3.3.2. 2-Hydroxy-5-methylbenzenesulfonic Acid (***2***) Formation Using Different Bases

1.2 g of chlorosulfonic acid (10 mmol) was added dropwise with constant stirring to an ice-cooled solution of 1 g of *p*-cresol (9.25 mmol) and a base (18.5 mmol; pyridine 1.5 mL or triethylamine 2.6 mL, or 2,6-lutidine 2.2 mL) in 10 mL of dichloromethane anhydrous. After 1 h, NaOH was added and the precipitate was recovered by filtration under vacuum. The solid was solubilized in methanol and reprecipitated with cold diethyl ether giving the sodium salt of compound **2** as a white solid (7.86–8.50 mmol, 85–92%). The same reactions were performed by adding the reagents in the following order: chlorosulfonic acid, base, and *p*-cresol. The title compound was obtained by treatment with acid under mild conditions.

### 3.4. NMR Analysis

NMR spectra were registered on a Bruker AVANCE 500 spectrometer equipped with a 5-mm broadband reverse probe and deuterium lock with field z-gradient operating at 500.13 and 125.76 MHz for ^1^H and ^13^C, respectively from 40 mM solutions (20 mmol of product dissolved in 0.5 mL of solvent). All NMR spectra were recorded at 298 K in D_2_O (isotopic enrichment 99.98%) solution and the chemical shifts were reported on a *δ* (ppm) scale. The proton spectra calibration of the chemical shift scale was performed by adjusting the residual H_2_O signals to 4.70 ppm (298 K, 40 mM, pH = 7.0). The chemical shifts of the carbon spectra in D_2_O were referenced to external TSP-d4 (3-(trimethylsilyl)-propionic-2,2,3,3-*d_4_* acid sodium salt) at 0.00 ppm. Acquisition parameters for 1D were as follows: ^1^H spectral width of 5000 Hz and 32 K data points providing a digital resolution of ca. 0.305 Hz per point, relaxation delay 2 s; ^13^C spectral width of 29412 Hz and 64 K data points providing a digital resolution of ca. 0.898 Hz per point, relaxation delay 2.5 s. The experimental error in the measured 1H-1H coupling constants was ±0.5 Hz.

#### 3.4.1. *p*-Cresyl Sulfate (pCS, **1**)

^1^H-NMR (D_2_O) δ: 7.03 (2H, d, *J* = 8.4 Hz, 3-H, 5-H), 6.71 (2H, d, *J* = 8.4 Hz, 2-H, 6-H), 2.14 (3H, s, CH_3_); ^13^C-NMR δ: 153.0 (C-1), 130.3 (C-3, C-5), 130.8 (C-4), 115.3 (C-2, C-6), 19.5 (CH_3_).

#### 3.4.2. 2-Hydroxy-5-methylbenzenesulfonic Acid (**2**)

^1^H-NMR (D_2_O) δ: 7.16 (1H, d, *J* = 2.4 Hz, 6-H), 6.82 (1H, dd, *J* = 2.4, 8.3 Hz, 4-H), 6.47 (1H, d, *J* = 8.3 Hz, 3-H), 2.28 (3H, s, CH3); ^13^C-NMR δ: 162.1 (C-2), 133.8 (C-4), 129.0 (C-5), 127.9 (C-6), 121.8 (C-1), 121.4 (C-3), 19.3 (CH_3_); mp. 250 °C.

### 3.5. Mass Spectrometry

All mass spectrometric measurements were performed in an AB Sciex 3200 QTRAP instrument with own electrospray ion source and proprietary software, and operated according to the manufacturer’s instructions. Measurements were performed either by infusion with the 1-mL instrument’s syringe pump (flow of 5 or 10 μL/min). For infusion, usually solutions of the compound at a final concentration of 1–10 ng/mL were used.

Source spectra were scanned in the negative ion mode between *m/z* 100 and 600, at a scan speed of 2 s per spectrum, in the multichannel analysis (MCA) mode, as the sum of 25 individual, consecutive scans.

Fragment ion spectra were recorded in the negative ion mode both in the conventional Q3-filter mode and in the Q3-trap (enhanced parent ion, EPI) mode. Recording started from *m/z* 50 to approximately 5 *m/z* higher than that of the precursor ion. Collision-induced dissociation was achieved with nitrogen, at the gas low pressure setting (approximately 2.5 × 10^−5^ Torr, with 0.8 × 10^−5^ Torr being the background gas pressure). In the conventional Q3-filter mode, a scan speed of 5 s per spectrum was employed. In the Q3-trap (enhanced parent ion, EPI) mode, scan speed was set at the slowest value of 250 amu/s, that which yields the best ion resolution. Integrated fragment ion spectra were recorded in both Q3 scan modes, using the ramp parameter. Briefly, one fragment ion spectrum was obtained at each value of the collision potential (CP) in an interval starting from 0 ΔV and up to 70 ΔV in 0.5- or 1-V increments. The data file was thus composed of a number of individual Q3 scans equal to the number of voltage steps of the ramp parameter function. From this data file, the ion current of each ion was extracted with the XIC command of the analyst software, and exported as a separate .txt file, that was imported in a Microsoft Excel spreadsheet for post-acquisition data elaboration.

All displayed measurements are obtained by post-acquisition data elaboration in a Microsoft Excel spreadsheet, essentially following literature [38,39,40], to measure integrated intensities of ions and their thermodynamic parameters such as appearance energies of fragments from the respective breakdown curves.

### 3.6. LC-MS/MS Conditions

Separation of synthesized *p*CS (**1**), 2-hydroxy-5-methylbenzenesulfonic acid (**2**), and standard IxS was carried out on HPLC Dionex 3000 UltiMate system (Thermo Fisher Scientific, MA, USA) coupled to the tandem mass spectrometer AB Sciex 3200 QTRAP (AB Sciex, Milan, Italy) operating under ESI ionization and in the negative ion mode. The instrument parameters were: CUR 30, GS1 40, GS2 50, capillary voltage −4.5 kV, and source temperature 550 °C. The three compounds were analyzed by selected reaction monitoring (SRM) checking the transitions: *m/z* 187.1 > 80 (−36 eV), 187.1 > 107 (−32 eV) for both *p*CS (**1**) and 2-hydroxy-5-methylbenzenesulfonic acid (**2**), whereas *m/z* 212.0 > 80 (−30 eV) and 212.0 > 132.1 (−26 eV) for IxS. The declustering potential was fixed to −40 ΔV. Chromatographic separation was achieved on an Accucore PFP 2.1 × 100 mm 2.6 μm (Thermo Fisher Scientific, MA, USA) using as mobile phase (A) 5 mM ammonium formate + 0.01% formic acid and (B) acetonitrile. The flow rate was 250 µL/min, and the column and the autosampler were held at 30 °C and 15 °C, respectively. The gradient elution was the following: 0–1 min 10% (B), 1–3.5 min 10–50% (B), 3.5–5.5 min 50% (B), and 5.5–6 min 50–10% (B) held until 8 min. Retention times were 2.7 min for both *p*CS (**1**) and 2-hydroxy-5-methylbenzenesulfonic acid (**2**) and 3.0 min for IxS.

## 4. Conclusions

This study starts from the awareness, given by NMR spectra, that initial synthetic approaches unexpectedly produced only 2-hydroxy-5-methylbenzenesulfonic acid (**2**), instead of the desired *p*CS (**1**) obtained following a different synthetic pathway. Possibly slight modifications of experimental procedure, gave one compound rather than the other. ^1^H and ^13^C magnetic resonance spectroscopy can distinguish the different molecular structures, since this technique is strongly sensitive to symmetry in the molecular connectivity. Interestingly the possibility to compare the two isomers **1** and **2** allowed us to realize that the two molecules are indistinguishable by mass spectrometry. Mass spectrometry even performed with the mildest ionization technique highlighted an unexpected loss of specific molecular connectivity in the formed ions because **1** and **2** show the same fragmentation pattern and in addition cannot be separated on several commercially available columns. The only difference between the two compounds is a 10-fold higher ionization yield under negative ion electrospray ionization. These considerations could be useful to avoid mistaking the compounds, bearing in mind that *p*CS (**1**), the protein-bound uremic retention solute, shall be considered in the sera of patients with chronic kidney disease rather than 2-hydroxy-5-methylbenzenesulfonic acid (**2**), that is not a human metabolite.

The observation that the ionization sensitivity of the two constitutional isomers **1** and **2** is different by a full order of magnitude could have consequences when levels of *p*CS are measured in clinical studies. The wrong isomer is much more ionization-prone that the natural metabolite, so if it is used to prepare analytical standards, measured levels are deceivingly much lower than the real ones, thus making mass balance unreliable.

## Supplementary Materials

The following are available online at https://www.mdpi.com/1420-3049/24/20/3704/s1, Figure S1. A: negative-ion fragment spectrum of pCS re-elaborated from Attygalle et al., 2001; center-of-mass collision energy 4.88 eV), B: Fragment spectrum of pCS (our measurement, center-of-mass collision energy 4.88 eV). Figure S2. Breakdown curves of the main ion species of pCS (1; A) and 2-hydroxy-4-methyl-phenylsulfonic acid (2; B). Figure S3. Possible mechanisms for the formation of the observed fragment ions in the spectra of: A) pCS (1) and B) 2-hydroxy-4-methyl-phenylsulfonic acid (2). Three further articles are cited as accompanying references.

## References

[1] Liabeuf S., Barreto D.V., Barreto F.C., Meert N., Glorieux G., Schepers E., Temmar M., Choukroun G., Vanholder R., Massy Z.A. (2010). Free p-cresylsulphate is a predictor of mortality in patients at different stages of chronic kidney disease. Nephrol. Dial. Transplant..

[2] Barreto F.C., Barreto D.V., Liabeuf S., Meert N., Glorieux G., Temmar M., Choukroun G., Vanholder R., Massy Z.A. (2009). Serum Indoxyl Sulfate Is Associated with Vascular Disease and Mortality in Chronic Kidney Disease Patients. Clin. J. Am. Soc. Nephrol..

[3] Lin C.-J., Chen H.-H., Pan C.-F., Chuang C.-K., Wang T.-J., Sun F.-J., Wu C.-J. (2011). p-cresylsulfate and indoxyl sulfate level at different stages of chronic kidney disease. J. Clin. Lab. Anal..

[4] Huang S.-T., Shu K.-H., Cheng C.-H., Wu M.-J., Yu T.-M., Chuang Y.-W., Chen C.-H. (2012). Serum Total p-Cresol and Indoxyl Sulfate Correlated With Stage of Chronic Kidney Disease in Renal Transplant Recipients. Transplant. Proc..

[5] Gryp T., Vanholder R., Vaneechoutte M., Glorieux G. (2017). p-Cresyl Sulfate. Toxins.

[6] Gabriele S., Sacco R., Altieri L., Neri C., Urbani A., Bravaccio C., Riccio M.P., Iovene M.R., Bombace F., De Magistris L. (2016). Slow intestinal transit contributes to elevate urinary p-cresol level in Italian autistic children. Autism Res..

[7] Wang C.-H., Cheng M.-L., Liu M.-H., Shiao M.-S., Hsu K.-H., Huang Y.-Y., Lin C.-C., Lin J.-F. (2016). Increased p-cresyl sulfate level is independently associated with poor outcomes in patients with heart failure. Heart Vessels.

[8] Stone R.W., Machamer H.E. (1947). Production of p-cresol and phenol from tyrosine by marine mud cultures. J. Bacteriol..

[9] Passmore I.J., Letertre M.P.M., Preston M.D., Bianconi I., Harrison M.A., Nasher F., Kaur H., Hong H.A., Baines S.D., Cutting S.M. (2018). Para-cresol production by Clostridium difficile affects microbial diversity and membrane integrity of Gram-negative bacteria. PLOS Pathog..

[10] Rajakovich L.J., Balskus E.P. (2019). Metabolic functions of the human gut microbiota: The role of metalloenzymes. Nat. Prod. Rep..

[11] Broderick J.B., Duffus B.R., Duschene K.S., Shepard E.M. (2014). Radical S-Adenosylmethionine Enzymes. Chem. Rev..

[12] Duranton F., Cohen G., De Smet R., Rodriguez M., Jankowski J., Vanholder R., Argiles A. (2012). Normal and Pathologic Concentrations of Uremic Toxins. J. Am. Soc. Nephrol..

[13] Meyer T.W., Hostetter T.H. (2012). Uremic solutes from colon microbes. Kidney Int..

[14] Ito S., Yoshida M. (2014). Protein-Bound Uremic Toxins: New Culprits of Cardiovascular Events in Chronic Kidney Disease Patients. Toxins.

[15] Viaene L., Annaert P., de Loor H., Poesen R., Evenepoel P., Meijers B. (2013). Albumin is the main plasma binding protein for indoxyl sulfate and p-cresyl sulfate. Biopharm. Drug Dispos..

[16] Pretorius C.J., McWhinney B.C., Sipinkoski B., Johnson L.A., Rossi M., Campbell K.L., Ungerer J.P.J. (2013). Reference ranges and biological variation of free and total serum indoxyl- and p-cresyl sulphate measured with a rapid UPLC fluorescence detection method. Clin. Chim. Acta.

[17] Schepers E., Meert N., Glorieux G., Goeman J., Van der Eycken J., Vanholder R. (2006). P-cresylsulphate, the main in vivo metabolite of p-cresol, activates leucocyte free radical production. Nephrol. Dial. Transplant..

[18] de Loor H., Meijers B.K.I., Meyer T.W., Bammens B., Verbeke K., Dehaen W., Evenepoel P. (2009). Sodium octanoate to reverse indoxyl sulfate and p-cresyl sulfate albumin binding in uremic and normal serum during sample preparation followed by fluorescence liquid chromatography. J. Chromatogr. A.

[19] Cuoghi A., Caiazzo M., Bellei E., Monari E., Bergamini S., Palladino G., Ozben T., Tomasi A. (2012). Quantification of p-cresol sulphate in human plasma by selected reaction monitoring. Anal. Bioanal. Chem..

[20] Watanabe H., Miyamoto Y., Enoki Y., Ishima Y., Kadowaki D., Kotani S., Nakajima M., Tanaka M., Matsushita K., Mori Y. (2015). p -Cresyl sulfate, a uremic toxin, causes vascular endothelial and smooth muscle cell damages by inducing oxidative stress. Pharmacol. Res. Perspect..

[21] Watanabe H., Miyamoto Y., Honda D., Tanaka H., Wu Q., Endo M., Noguchi T., Kadowaki D., Ishima Y., Kotani S. (2013). p-Cresyl sulfate causes renal tubular cell damage by inducing oxidative stress by activation of NADPH oxidase. Kidney Int..

[22] Watanabe H., Noguchi T., Miyamoto Y., Kadowaki D., Kotani S., Nakajima M., Miyamura S., Ishima Y., Otagiri M., Maruyama T. (2012). Interaction between Two Sulfate-Conjugated Uremic Toxins, p-Cresyl Sulfate and Indoxyl Sulfate, during Binding with Human Serum Albumin. Drug Metab. Dispos..

[23] Feigenbaum J., Neuberg C.A. (1941). Simplified Method for the Preparation of Aromatic Sulfuric Acid Esters. J. Am. Chem. Soc..

[24] Deno N.C., Newman M.S. (1950). Mechanism of Sulfation of Alcohols 1,2. J. Am. Chem. Soc..

[25] Dado G., Bernhardt R. (2017). Sulfonation and Sulfation. Kirk-Othmer Encyclopedia of Chemical Technology.

[26] Suter C.M., Oberg E. (1934). A Quantitative Study of the Reaction between Some Primary Aliphatic Alcohols and Sulfuric Acid. J. Am. Chem. Soc..

[27] Cerfontain H., Koeberg-Telder A., Lambrechts H.J.A., De Wit P. (1984). Aromatic sulfonation. 90. Sulfonation of three symmetrical 2,6-dialkylphenols, 2,6-dimethylanisole. Sulfation and sulfonation product distributions and mechanisms. J. Org. Chem..

[28] Cerfontain H., Koeberg-telder A. (1989). Sulfonation and sulfation in the reaction of 4-methylphenol with concentrated sulfuric acid. Phosphorus. Sulfur. Silicon Relat. Elem..

[29] Goossens H.D., Lambrechts H.J.A., Cerfontain H., de Wit P. (2010). Sulfonation and sulfation in reactions of C-methylated phenols and anisoles with sulfur trioxide. 4-Substituted phenyl hydrogen sulfates: Effective reagents for transsulfonation. Recl. Des Trav. Chim. Des Pays-Bas.

[30] Ragan M.A. (1978). Phenol sulfate esters: Ultraviolet, infrared, 1 H and 13 C nuclear magnetic resonance spectroscopic investigation. Can. J. Chem..

[31] Karimi-Jaberi Z., Poolodian B., Moradi M., Ghasemi E. (2012). 1,3,5-Tris (hydrogensulfato) Benzene: A New and Efficient Catalyst for Synthesis of 4,4′-(arylmethylene)bis(1H-pyrazol-5-ol) Derivatives. Chin. J. Catal..

[32] Duffel M.W., Marshall A.D., McPhie P., Sharma V., Jakoby W.B. (2001). Enzymatic aspects of the phenol (aryl) sulfotransferases. Drug Metab. Rev..

[33] Ciuffreda P., Brizzolari A., Casati S., Eberini I., Palazzolo L., Parravicini C., Santaniellob E. (2016). 2,4-Furfurylidene-D-sorbitol and its tetra-methyl ether: Synthesis, conformational studies, and radical scavenging activity. Arkivoc.

[34] Ciuffreda P., Casati S., Santaniello E. (2000). The action of adenosine deaminase (E.C. 3.5.4.4.) on adenosine and deoxyadenosine acetates: The crucial role of the 5’-hydroxy group for the enzyme activity. Tetrahedron.

[35] Attygalle A.B., García-Rubio S., Ta J., Meinwald J. (2001). Collisionally-induced dissociation mass spectra of organic sulfate anions. J. Chem. Soc. Perkin Trans..

[36] Casati S., Manzocchi A., Ottria R., Ciuffreda P. (2010). 1H, 13C and 15N NMR assignments for N6-isopentenyladenosine/inosine analogues. Magn. Reson. Chem..

[37] Casati S., Manzocchi A., Ottria R., Ciuffreda P. (2011). 1H, 13C and 15N NMR spectral assignments of adenosine derivatives with different amino substituents at C6-position. Magn. Reson. Chem..

[38] Chinthakindi P.K., Rath S.K., Singh J., Singh S., Koul S., Sangwan P.L. (2017). Isolation of isoxanthanol and synthesis of novel derivatives as potential cytotoxic agents. Med. Chem. Res..

[39] Rubino F.M., Pitton M., Caneva E., Pappini M., Colombi A. (2008). Thiol-disulfide redox equilibria of glutathione metaboloma compounds investigated by tandem mass spectrometry. Rapid Commun. Mass Spectrom..

[40] Rubino F.M., Pitton M., Brambilla G., Colombi A. (2006). A study of the glutathione metaboloma peptides by energy-resolved mass spectrometry as a tool to investigate into the interference of toxic heavy metals with their metabolic processes. J. Mass Spectrom..

