# Supplementary material for: Unambiguous Characterization of p-Cresyl Sulfate, a Protein-Bound Uremic Toxin, as Biomarker of Heart and Kidney Disease
Source: Molecules. 2019 Oct 15;24(20):3704. doi: 10.3390/molecules24203704 (PMC6832250; doi:10.3390/molecules24203704)
Supplement: Supplementary file 1 [file molecules-24-03704-s001.pdf]

Supplemental Material

Unambiguous characterization of p-cresyl sulfate, a protein-bound uremic toxin, as biomarker of heart and kidney disease

Rita Paroni [a*], Silvana Casati[b*], Michele Dei Cas[a], Monica Bignotto[a] Federico Maria Rubino[a], Pierangela Ciuffreda[b#]

[a] Dipartimento di Scienze della Salute, Università degli Studi di Milano, Via Di Rudinì, 8, 20142 Milano, Italy rita.paroni@unimi.it, michele.deicas@unimi.it, monica.bignottonimi.it, federico.rubino@unimi.it

[b] Dipartimento di Scienze Biomediche e Cliniche "Luigi Sacco", Università degli Studi di Milano, Via G.B. Grassi 74, 20157 Milano, Italy silvana.casati@unimi.it, pierangela.ciuffreda@unimi.it

* Both authors contributed equally to this manuscript

[#] Correspondence: pierangela.ciuffreda@unimi.it; Tel: +39-02-50319695

Molecules 2019 xxx

Table of Contents

Figures

Figure S1. A: negative-ion fragment spectrum of pCS re-elaborated from Attygalle et al., 2001; center-of-mass collision energy 4.88 eV), B: Fragment spectrum of pCS (our measurement, center-of-mass collision energy 4.88 eV). Pag. 2

Figure S2. Breakdown curves of the main ion species of pCS (1; A) and 2-hydroxy-4-methyl-phenylsulfonic acid (2; B). Pag. 2

Figure S3. Possible mechanisms for the formation of the observed fragment ions in the spectra of: A) pCS (1) and B) 2-hydroxy-4-methyl-phenylsulfonic acid (2). Pag. 3

References Pag. 3

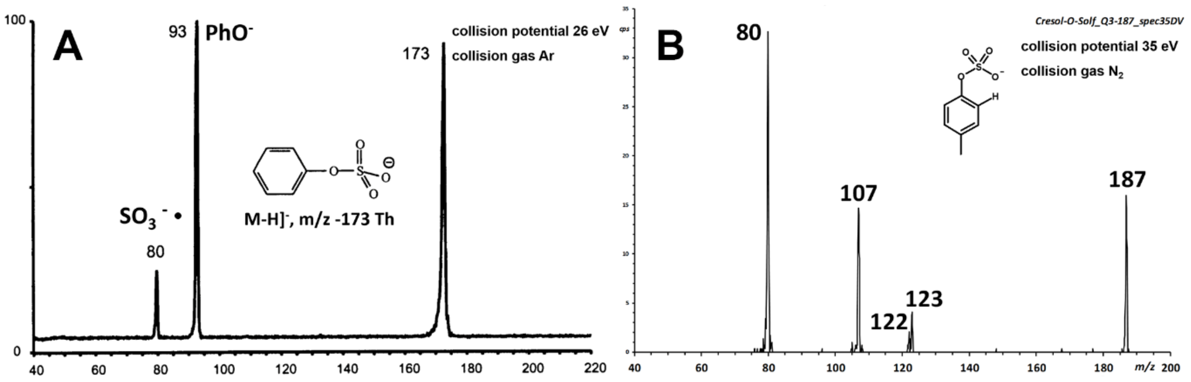

Figure S1. A: negative-ion fragment spectrum of *p*CS re-elaborated from Attygalle *et al*., 2001; center-of-mass collision energy 4.88 eV), **B:** Fragment spectrum of *p*CS (our measurement, center-of-mass collision energy 4.88 eV).

The spectrum reported by Attygalle *et al*. (2001) was recorded at 27 ΔV collision energy on Argon target gas. To improve comparison, one was purposely recorded at the same value of "effective" center-of-mass collision energy in our instrument that employs Nitrogen as target gas.

In addition, unimolecular decomposition of the deprotonated molecule (*m*/*z* 187) of both compounds yields almost superimposable breakdown curves of the main fragment ions (Figure S2).

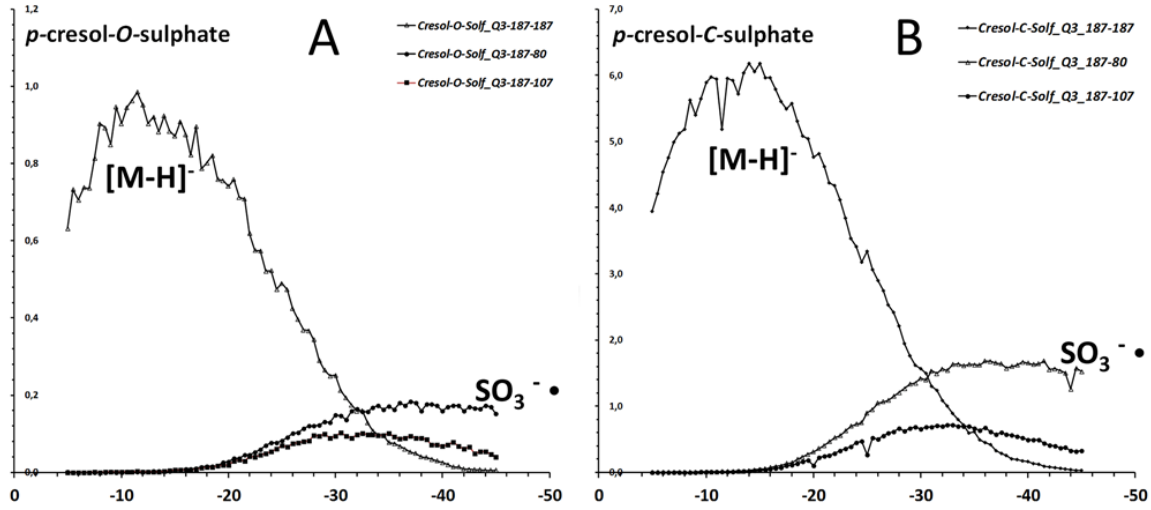

Figure S2. Breakdown curves of the main ion species of *p*CS (**1**; A) and 2-hydroxy-4-methyl-phenylsulfonic acid (**2**; B).

According to the fragmentation schemes of the two isomers reported as Figure S3, possible connectivities indicated as (I) and (II) may be generated from either isomers, and evolve to yield all four fragment ions. The three main transitions onset and climax at essentially the same value of collision energy (onset: approx. 17 ΔV, corresponding to approx. 2.2 eV; climax: approx. 32 ΔV, corresponding to approx. 4.2 eV). This unexpected behavior, and especially decomposition starting at a so high value of excess energy, suggests that the two isomers may convert in the gas-phase to a common structure that "bursts" into the two main fragments, deprotonated *p*-cresol and electron-attached sulfur trioxide (Electron affinity of SO_3 is 43.8 kcal/mol or 1,8993 eV; [1]).

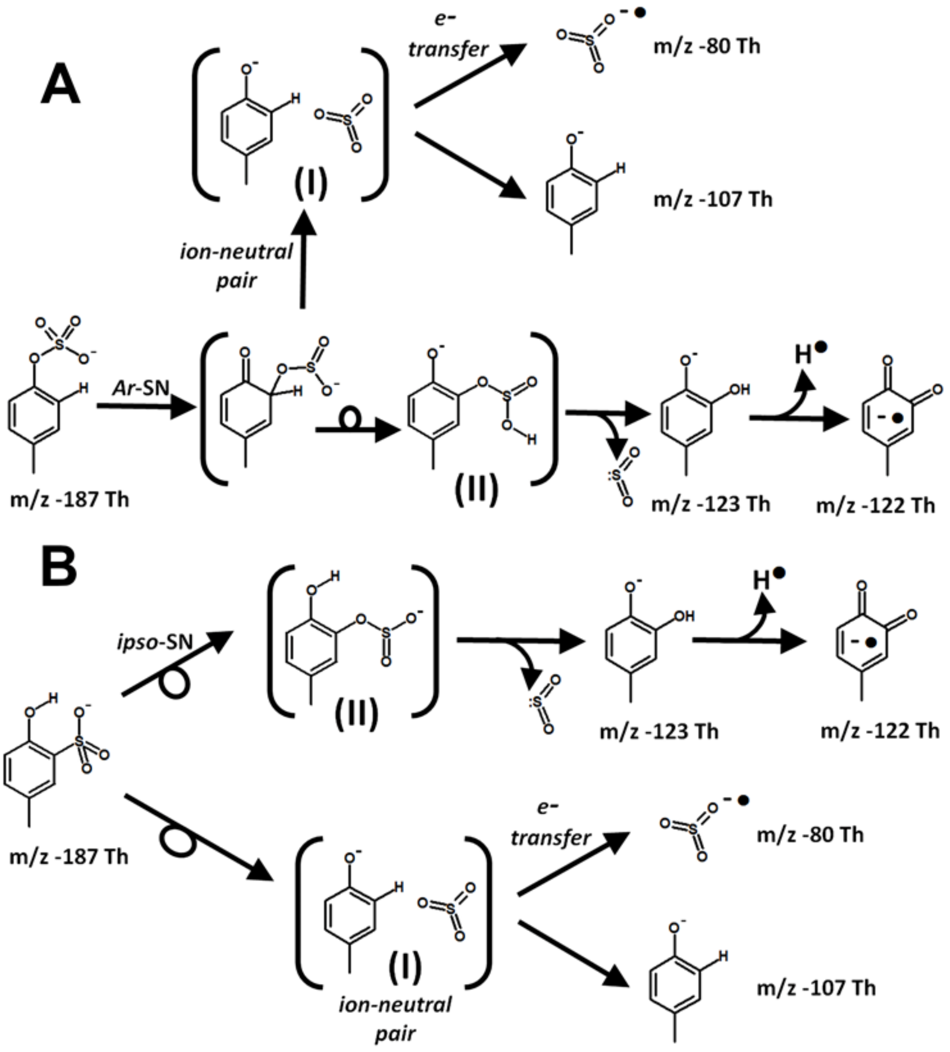

Figure S3. Possible mechanisms for the formation of the observed fragment ions in the spectra of: A) *p*CS (**1**) and B) 2-hydroxy-4-methyl-phenylsulfonic acid (**2**).

The minor reaction that leads to m/z 123 can be envisaged as the formal disproportionation to deprotonated 4-methyl-hydroquinone and sulfur dioxide. It is intriguing to note that oxygen insertion from the >SO2 of sulphonyl groups in the ipso- position of linked aromatic systems is also observed in some aryl-sulfonamides of alpha- and beta- amino-acids (Rubino FM, unpublished), and possibly shares a common mechanism with the frequent SO_2 extrusion from aromatic sulfonamides [2, 3]. This unexpected phenomenon that occurs by ion generation with the mildest available ionization technique suggests that isomerization of both compounds to a common non-covalently bound connectivity likely occurs already at the desolvation stage of the ESI mechanism. At the best of mass spectrometric insight, thus, the two isomers cannot be discriminated.

References

1. H. P. Gunawardena et al., "Electron Transfer versus Proton Transfer in Gas-Phase Ion/Ion Reactions of Polyprotonated Peptides," J. Am. Chem. Soc., vol. 127, no. 36, pp. 12627–12639, Sep. 2005.

2. G. Hopfgartner, W. Vetter, W. Meister, and H. Ramuz, "Fragmentation of Bosentan ® (Ro 47-0203) in ionspray mass spectrometry after collision-induced dissociation at low energy: a case of radical fragmentation of an even-electron ion," J. Mass Spectrom., vol. 31, no. 1, pp. 69–76, Jan. 1996.

3. Z. Wang, C. E. C. A. Hop, M.-S. Kim, S.-E. W. Huskey, T. A. Baillie, and Z. Guan, "The unanticipated loss of SO_2 from sulfonamides in collision-induced dissociation," Rapid Commun. Mass Spectrom., vol. 17, no. 1, pp. 81–86, Jan. 2003.
